# An Attempt to Conceptualize the Phenomenon of Stigma toward Intimate Partner Violence Survivors: A Systematic Review

**DOI:** 10.3390/bs13030194

**Published:** 2023-02-21

**Authors:** Federica Taccini, Stefania Mannarini

**Affiliations:** 1 Interdepartmental Center for Family Research, University of Padova, 35131 Padova, Italy; 2 Department of Developmental Psychology and Socialization, University of Padova, 35131 Padova, Italy; 3 Department of Philosophy, Sociology, Education, and Applied Psychology, Section of Applied Psychology, University of Padova, 35131 Padova, Italy

**Keywords:** intimate partner violence, stigma, public stigma, self-stigma, domestic violence, clinical psychology

## Abstract

The objectives of the present manuscript were to review the literature on stigma toward survivors of intimate partner violence (IPV) and to identify the most widely used assessment techniques to investigate this issue. The PRISMA guidelines were followed, and the systematic review was registered in PROSPERO, registration number: CRD42022327410. PsycINFO, Scopus, Web of Science, and PubMed were searched. Two authors selected and extracted data from eligible studies. In total, 4220 hits were returned from the database search, and of them, 24 articles met the inclusion criteria. The articles included in the review confirm the presence of stigma toward IPV survivors, which can be divided into public stigma and self-stigma. Specifically, 17 studies were related only to public stigma, 1 study focused only on self-stigma, and 6 articles investigated aspects related to both public and self-stigma. Both qualitative and quantitative studies have been conducted on this topic. The considerations on the methodologies and assessment measures used in the included studies will be discussed in the results section. Based on the research included, it was possible to develop a contribution to the definition of stigma, which will be discussed in the article.

## 1. Introduction

Intimate partner violence (IPV) corresponds to any behavior and conduct a person exerts against the partner that inflicts physical, psychological, or sexual harm [1]. Globally, around 30% of women are physically or sexually abused by a partner [2,3]. Considering IPV’s global diffusion, cultural factors must be taken into account when addressing this phenomenon. In fact, culture can play a key role in increasing or decreasing the risk of IPV and its associated outcomes, for example, by legitimizing violence in intimate relationships and by attributing a passive role to women [4,5,6].

Experiencing IPV contributes to the development of PTSD, depression, anxiety, substance abuse, and suicide attempts [7,8,9,10,11,12,13]. In addition, IPV survivors can experience another kind of victimization: Stigma [14]. Stigma is “a multileveled term alternately representing the cues or marks that signal stereotypes and prejudice, and the rubric representing the overall stereotypical and prejudicial process” [15] (p. 51). Consequently, it can be considered an umbrella term that includes stereotypes, prejudices, and discriminatory behaviors against a group of individuals [16,17,18]. Stereotypes have been defined as negative beliefs about a group of people [19], while the term prejudice refers to the emotional response that results from the acceptance of a stereotype [15,19,20]. Lastly, the behavioral implication of prejudice corresponds to discrimination [15,19,20]. Most research in the field has focused on stigma toward mental health, e.g., [21,22]. However, stigma affects different social groups, including IPV survivors [14,23,24].

Previous research has distinguished between public and self-stigma [15,19,20]. On the one hand, public stigma “comprises reactions of the general public towards a group based on stigma about that group” [25] (p. 530). Public stigma leads to the experience of everyday discrimination, ostracism, and professional inaction [26]. Regarding IPV survivors, they can experience victim-blaming attitudes—when the survivor is blamed for the violence she has been subjected to [27]—and secondary victimization—which corresponds to victim-blaming conducts and attitudes acted out by community service providers that cause additional trauma for survivors [28]. Consequently, public stigma can affect the help-seeking process of IPV survivors [14]. In addition to this, same-sex couples may face a wide range of specific prejudices, stereotypes, and negative feelings toward homosexuality—homophobia [29,30,31,32].

On the other hand, self-stigma is described as “the reactions of individuals who belong to a stigmatized group and turn the stigmatizing attitudes against themselves” [25] (p. 531). Experiencing this type of stigma results in feelings of shame and blame [25,33,34]. These feelings can affect the help-seeking process [25] and people’s self-esteem and self-efficacy [25,35].

The present systematic review aims to investigate stigma toward female IPV survivors based on the conceptualization of stigma mentioned above. Specifically, the research questions are the following: What is the state-of-the-art stigma against IPV survivors and its effects on them? What are the most widely used measures and assessment techniques implemented in the literature to address this issue?

## 2. Materials and Methods

### 2.1. Search Strategy

This review followed the Preferred Reporting Items for Systematic Reviews and Meta-analyses (PRISMA statement) guidelines [36]. The PRISMA checklists [37] for this review are provided in the Supplementary Material. The systematic review is registered in the PROSPERO database of systematic reviews (https://www.crd.york.ac.uk/prospero/ (accessed on 15 February 2023); registration number: CRD42022327410. The search strategy has been conducted in the following electronic databases: Web of Science (WOS), Scopus, PubMed, and PsycINFO. The keywords included are the following: (intimate partner violen* OR domestic violen* OR domestic abus* OR Spous* abus* OR spous* violen*) AND (stigm* OR public stigm* OR self-stigm* OR discrimin* OR prejud* OR victim blam* OR second* victim* OR blam* OR stereotyp*).

### 2.2. Inclusion and Exclusion Criteria

Manuscripts were included when they: (a) Were published in a peer-reviewed journal between January 2011 and December 2021, (b) were written in English or Italian, (c) concerned research conducted in an English or Italian speaking western country, (d) concerned the phenomenon of stigma toward female victims of intimate partner violence by a male partner in adulthood (>18 years old) both from a general population perspective and a survivor perspective.

Manuscripts were excluded when: (a) They were unpublished manuscripts and thesis, reviews, commentaries, editorials, conference proceedings, conference paper, meeting abstract, book chapter or opinion pieces, case reports, randomized controlled trial, (b) they did not include participants (i.e., general population, victims of violence) therefore, manuscripts concerning analysis of newspapers, posts/comments, etc., on social media, of archival data, of trial transcripts were excluded, (c) they were written in languages other than English and Italian, (d) articles that did not explicitly investigate the stigma or one of the following aspects of this phenomenon—which are stigm*, public stigm*, self-stigm*, discrimin*, prejud*, victim blam*, second* victim*, stereotyp*—in the objectives/hypotheses of the research have been excluded, (e) articles in which the constructs and the investigated phenomenon were not defined, (f) articles that did not focus only on intimate partner violence, but also investigated other typologies of violence.

### 2.3. Data Extraction

Study selection was a two-stage process: First, studies were selected on the basis of their titles and abstracts. Then, the two authors independently evaluated the full text of the potentially eligible studies. A standardized Excel form was used to extract data from eligible studies to assess their quality and evidence synthesis. This form includes authors, year of publication, aim/hypothesis, any secondary aims, type of stigma investigated, study design, measures, sample size, study population characteristics, comparison group (if present), sample size, methodology, measures, methodology and statistical analyses, results, conclusions, limitations of the study, limitations of the methodology, study risk of bias, strengths of the study, strengths of the measures, key words used, electronic database, date, notes, study included after screening, reason for exclusion during screening. Any disagreement between the reviewers was resolved by consensus.

### 2.4. Identified Studies

**Figure 1:**
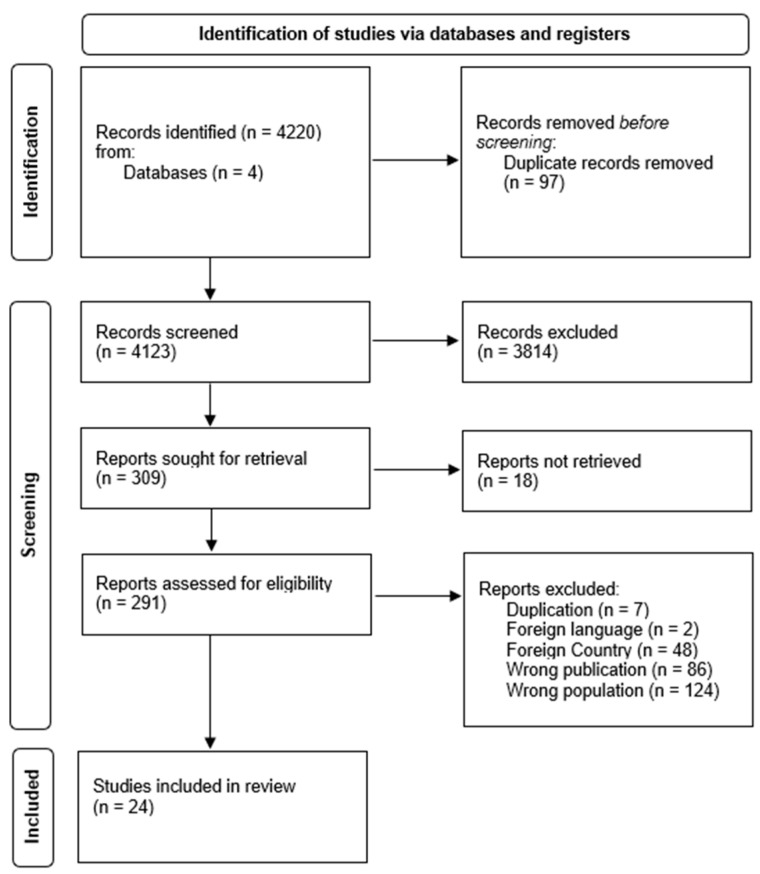
PRISMA Flow Diagram.

In total, 4220 hits were returned from the database search (see Figure 1). Then, all duplicates were removed (*n* = 97), which resulted in a sample of 4123 publications. The titles and abstracts of these publications were independently screened by two reviewers, FT and SM, and resulted in a sample of 309 articles ready for full text screening. However, 18 articles were not retrieved since it was not possible to access their full report. Consequently, the resulting number of articles assessed for eligibility was 291. However, 267 of them were excluded because they were duplicates or did not meet the inclusion criteria. The resulting sample of included articles is 24, that all met the quality assessment criteria (see Figure 1 and Table 1).

## 3. Results

### 3.1. Defining Stigma toward IPV Survivors

This systematic review confirms that IPV survivors experience stigma in their daily lives. However, the 24 articles included in this review (see Table 1) show that a shared definition of stigma toward IPV survivors is missing.

By summarizing all the results of the studies included in the present review and applying previous theories on stigma referred to mental illness [15,19,20,66], it is possible to assume that even with this population, the definition of stigma as a phenomenon that includes marks and stereotypes that lead stigmatized individuals to experience prejudice and discrimination against them appears to be confirmed [15,16,19,20,66].

IPV survivors seem to be stigmatized when they do not have the following characteristics of ‘ideal victims’: (1) Weak, (2) they were doing something respectable when the violent episode occurred, (3) they cannot be held responsible for the circumstances in which the violence took place, (4) they faced bigger offenders, (5) they faced unknown perpetrators [56,67].

Furthermore, stereotypes about IPV survivors appear to correspond to domestic violence/IPV myths, which are “misconceptions and false beliefs about intimate partner violence, victims, and abusers” [33] (p. 330). The most common among these are the following: The survivor is held responsible for the abuse, the violence that occurs is trivialized, the perpetrator is somehow justified, the IPV is assumed exclusively to be in the form of physical abuse [33,42,46,65].

Prejudices against IPV survivors appear to be related to feelings of blame, shame, and fear. In fact, the general population seems to blame IPV survivors for the violence they experienced. IPV survivors can internalize this blame and begin to consider themselves responsible for the abuse received while simultaneously being ashamed of it [27,28,33,53]. For example, Meyer [53] showed that 64.3% of their sample of IPV survivors reported previous experiences with victim blame attitudes and the need to demonstrate to both formal and informal supporters that they were not responsible for their own victimization. In addition, these women feared blame and discriminatory behaviors [52,68].

Discrimination against IPV survivors appears to correspond to secondary victimization that women can experience in their interpersonal interactions [49]. Secondary victimization corresponds to “the victim-blaming attitudes, behaviors, and practices engaged in by community service providers, which result in the additional trauma for rape survivors” [69] (p. 56). An example of this is the difficulty faced by survivors when trying to speak in the courtroom [28,49,69]. Rivera and colleagues [28] showed that 63% of the participants reported previous experiences of secondary victimization characterized by feelings of blame, of being disbelieved or dismissed, and consequently, 67% of these women did not want to return to court because they did not feel safe, believed, and respected.

### 3.2. Public Stigma

Twenty-three studies investigated public stigma in IPV survivors (see Table 1). Most of these studies have shown the presence of stigmatizing attitudes toward IPV survivors, which contributed to the development of mental health problems, such as depression and PTSD symptoms [14,39,48,64]. In this sense, examples of negative public reactions are victim blame, shame, and discrediting attitudes [14].

Yamawaki and colleagues [65] showed the presence of victim-blaming attitudes in undergraduate students, and these results have been confirmed in other studies [33,42]. In contrast, Eigenberg and Policastro [27] showed that most of their participants were unwilling to blame survivors of abuse.

Stigmatizing attitudes appeared in formal and informal supporters as well: Gutowski and Goodman [49] showed that most of the women in their study reported experiencing harsh behaviors and judgments from court professionals. In this regard, Nikolova and colleagues [56] confirmed these results with respect to advocates. Despite this, hand therapists have shown low levels of victim-blaming attitudes [60]. This result is encouraging, considering the high possibility that survivors of physical or sexual abuse attend hand therapy clinics [60].

A factor that appears to play a key role in public stigma is gender: Men endorsed victim-blaming attitudes more often, compared to women [26,27,33,38,42,45,58,65]. Furthermore, Yamawaki and colleagues [65] have shown that male undergraduate students tended to trivialize IPV episodes more often than females.

Another factor that appears to affect public stigma is the type of relationship the woman has with the offender. Yamawaki and colleagues [65] showed that participants tended to blame the survivor more when she was dating the abuser compared to when she was married to him. Furthermore, the decision to stay or return to the abusive partner led to increased levels of victim blame [50,58,65].

Another factor that seems to play a key role in public stigma is the adhesion to sexist, patriarchal, or conservative values [26,51,58,65]. For example, Riley and Yamawaki [58] showed that people with higher values of benevolent sexism (BS) reported that they would not have provided any support to women who do not leave the abuser.

In conclusion, few studies showed no public stigma toward female IPV survivors [39,48,60]. For example, Dardis and colleagues [39] reported that women who revealed their IPV experience received more positive than negative social reactions.

### 3.3. Self-Stigma

Six studies showed the presence of self-stigma in IPV survivors (see Table 1). With regard to this, self-stigma seems to have connotations of guilt and shame. Flicker and colleagues [48] showed the presence of a self-blame coping strategy along with denial and disengagement in women seeking a protection order against an abusive partner. Furthermore, self-stigma appeared to contribute to the development of depressive and post-traumatic symptoms [48,54,63]. Additionally, the experience of judgmental responses from figures who should be supportive instead (e.g., lawyers, police officers, etc.) negatively affected the psychological well-being of survivors: Srinivas and DePrince [63] showed that unmet police expectations significantly predicted the severity of PTSD symptoms. Furthermore, IPV survivors who experienced stigmatizing attitudes from courtroom professionals reported feeling ashamed, worthless, or powerless [49]. In contrast, the supportive social network acted as a protective factor against the development of psychological disorders [48,63].

Furthermore, interiorizing public blame for their experience of violence and a related feeling of shame can hinder the help-seeking process [68] and the choice to interrupt the relationship with the perpetrator [68]. Furthermore, the internalization of patriarchal gender roles could also play a role in this matter [52]: Women who have internalized gender stereotypes can struggle to act against them and break their abusive relationships [51].

### 3.4. Researching Stigma toward Survivors of IPV

Both qualitative and quantitative studies have investigated stigma toward IPV survivors.

The qualitative studies included in the present review implemented semi-structured [28,53,54,63,64] and in-depth interviews [51]. Some of the methodologies and approaches used for the interviews were: The phenomenological approach [51], the life history approach [54], the analytic method [49]. Two studies have also included a focus group [51,54]. Qualitative research allows one to investigate the first-person experience, which results in a deep understanding of human behavior [70]. In this field, to the best of our knowledge, only interviews and focus groups have been conducted.

Concerning the quantitative studies included in the present review, four of them used the vignette methodology to investigate public perceptions of IPV survivors [38,50,52,65]. Furthermore, the following self-reports were used in the research included in the present review: The Social Reactions Questionnaire (SRQ; Ullman [40]), which focused on social responses to the disclosure of survivors of violence. Survivors had to report how often they received 48 different reactions from other people to their disclosure. The instrument had good validity and reliability [40].

The Domestic Violence Myth Acceptance Scale [DVMAS; Peters [45]], which is an 18-item questionnaire that measures the endorsement of domestic violence myths by participants. The reliability of the questionnaire is excellent and presents good validity in terms of face and content. However, divergent validity was only partially supported in the study by Peters [46].

The Supportive Attitudes Toward Victim Scale (SAVS) is a 15-item questionnaire developed by Riley and Yamawaki [58] to assess the degree to which a person has supportive attitudes toward a survivor of violence. A principal component analysis reported the presence of four subscales: Insisting Victim to Leave subscale (α = 0.76), Imposing Judgment subscale (α = 0.75), Traditional Value for Intimate Relationships subscale (α = 0.69), and Work Out Relationship (α = 0.60).

The Domestic Violence Blame Scale (DVBS) [55] is a 23-item scale that aims to assess the amount of blame an individual attributes to survivors of domestic violence. According to Bryant and Spencer [55], it presents adequate reliability and validity.

The Victim Blame Attribution Scale (VBA) [59] investigates victim-blame attitudes. VBA has five items, and Cronbach’s alpha was 0.73 for an American population.

The Perceived Seriousness of Violence measure is a 5-item questionnaire aimed at assessing the degree to which individuals perceive the severity of IPV episodes [59]. Cronbach’s alpha was 0.84.

The Excuse Perpetrator Measure [56] is a 4-item questionnaire that evaluates the excusing of the batterer of the respondents. The Cronbach alpha was 0.58.

The Physician Readiness to Manage Intimate Partner Violence Survey (PREMIS) is a questionnaire designed to assess the self-perceived readiness of physicians [71] or students [61] to handle patients with a history of violence. The questionnaire is reliable and valid, and it allows one to discriminate between trained from non-trained physicians.

A questionnaire was designed to evaluate the attitudes, beliefs, and behaviors of health care providers about IPV recognition and handling [61]. The questionnaire consists of 39 items with good internal reliability (*α* = 0.88).

The Attitudes Toward Crime Victims index assesses law personnel’s beliefs about survivors of violent crimes’ attitudes and behaviors [45,47]. The questionnaire showed high internal reliability (*α* = 0.822).

To the authors’ knowledge, four other questionnaires have been developed to investigate aspects of public stigma toward IPV survivors. Articles related to the validation of these questionnaires did not meet the inclusion criteria of the present review, however, they may be useful to those conducting research in this field. The questionnaires are the following: Victim-Blaming—Intimate Partner Violence Against Women Scale [72], Acceptability of Intimate Partner Violence Against Women-8 Scale [73], Willingness to Intervene in Cases of Intimate Partner Violence Scale [74]. Moreover, another questionnaire has been developed to investigate self-stigma: The intimate partner violence stigma scale [75].

## 4. Discussion

The current study aimed to assess stigma toward female IPV survivors using a systematic review. Twenty-four studies were included in the review and confirmed the feasibility of the definition of stigma as a phenomenon that includes stereotypes that lead the stigmatized group to experience prejudice and discrimination [15,16,19,20,66]. In the case of IPV, stereotypes appear to refer to myths of domestic violence/IPV that are shared by the general population and consist of blaming survivors, minimizing violence, justifying the perpetrator, and considering IPV as corresponding only to physical abuse [28,33,42,46]. Additionally, prejudices appear to correspond to feelings of blame, shame, and fear in IPV survivors, perpetrators, and the general population. In fact, the general population seems to blame IPV survivors for the violence they experienced, and survivors can internalize this blame—self-stigma [27,28,33,53]. Lastly, the discrimination faced by survivors of violence in the course of their interpersonal interactions appears to correspond to secondary victimization [28,49].

**Figure 2:**
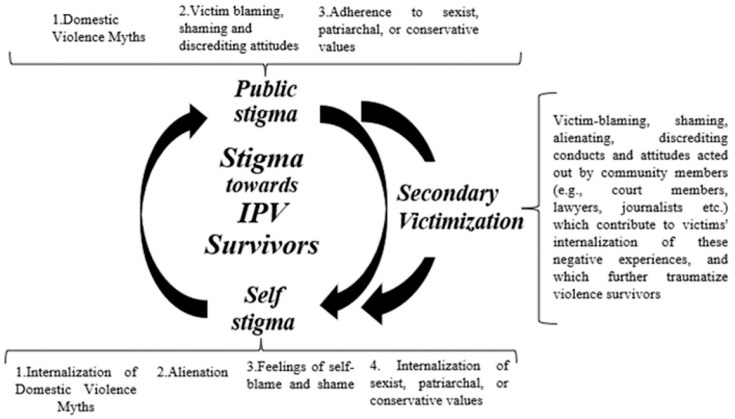
Definition of stigma towards IPV survivors.

The results show that women experience both public and self-stigma and that the two are intertwined for this population. Specifically, the experience of public stigmatizing attitudes contributes to the internalization of these negative experiences—self-stigma—which can also reinforce public stigma, resulting in a vicious circle that can be difficult to break (see Figure 2). In this cycle, secondary victimization also seems to play an important role. In fact, secondary victimization seems to contribute to the development of self-stigma in IPV survivors, as it constitutes a further form of public victimization and traumatization. It seems that through the discriminatory conducts typical of secondary victimization, a survivor could internalize the public stigma suffered and start adhering to such stigmatizing attitudes, and self-stigma.

Both public and self-stigma appear to have negative implications for the psychological well-being of survivors. IPV survivors reported depression and post-traumatic symptoms related to the stigma suffered [39,41,48,64]. Furthermore, stigma appears to affect the survivors’ help-seeking process and the support offered by formal and informal supporters as well. In fact, only a few studies showed that participants experienced more positive than negative social reactions to their disclosure [39,48,60].

Research in this field has been carried out through qualitative and quantitative studies. In this regard, several previously mentioned self-reports can allow researchers to investigate important components of stigma toward IPV survivors, e.g., [55,59]. However, to our knowledge, a questionnaire that addresses all aspects of public stigma toward IPV survivors is lacking. In addition, only one questionnaire investigates self-stigma [75].

### Implications for Practice, Intervention, and Policy

This systematic review presents important implications for intervention. Regarding self-stigma, women stressed their desire to overcome this barrier [53]. Consequently, psychological and social support for IPV survivors should focus on this issue [53]. Previous literature shows the benefits of targeting internalized stigma in the clinical setting [76,77,78]. In fact, psychological interventions focused specifically on this aspect appear to improve patients’ levels of empowerment, self-esteem, self-efficacy, and functioning in post hoc analyses [76,77,78,79]. Consequently, psychological and social support for IPV survivors should also focus on this issue [53].

Furthermore, anti-stigma prevention campaigns that aim to promote a non-stigmatizing culture must be developed to target public stigma [51]. In this regard, Keller and Honea [51] presented six tips that could be used when designing this kind of intervention: (1) Avoidance of stereotypical gender messages, (2) IPV should not be minimized, (3) survivors should not be blamed, (4) personal and social support for both batterers and survivors should be addressed, (5) access to IPV resources for the general population should be promoted, (6) a community-based approach should be used.

Additionally, formal supporters (such as police force, lawyers, etc.) should be trained on how to properly manage IPV cases. In this regard, Fleming and Franklin [45] showed that IPV training resulted in higher levels of preparedness and self-confidence in responding to family violence cases in members of the police force.

This review presents some limitations. First, the present review focused only on intimate partner violence. However, the literature also shows the presence of stigma against survivors of other types of violence (such as rape), e.g., [80,81]. Second, the present research aimed to investigate male-perpetrated violence against a female partner. However, the literature has shown the presence of partner abuse acted against male partners and in LGBT couples [82,83]. The stigmas these survivors face can take on different aspects that need to be explored in future studies. Third, the focus of the present review was on studies from 2011 onwards. This year was chosen due to its historical relevance for women’s rights with the Istanbul Convention [84], an international convention that defined a framework to protect women from all forms of violence. However, articles before that date are not included in this review. Fourth, the objective of the present review was to develop a comprehensive picture of the stigma against female IPV survivors in the western world. However, articles related to other countries were not included and therefore, should be addressed in future studies. Lastly, most studies investigated the phenomenon of public stigma. Consequently, more research on self-stigma should also be done to better understand this phenomenon. Furthermore, other qualitative methodological approaches, such as participatory action research, could be implemented in this regard, and psychological interventions targeted specifically at the self-stigma experienced by IPV survivors should be developed.

## 5. Conclusions

This systematic review stresses the relevance of the stigma phenomenon against IPV survivors, which affects their psychological well-being, their safety, and hinders their help-seeking process.

Therefore, it is important to develop psychological interventions with IPV survivors that aim to overcome the negative implications of stigma. Moreover, there seems to be a need for the development of anti-stigma campaigns aimed at promoting a non-stigmatizing culture.

## Figures and Tables

**Table 1:** Study characteristics.

Study	Participants	Type of Stigma	Researching Stigma	Selected Findings
Bothamley and Tully (2017) [38]	168 participants (31% were men, 69% were women)	Public stigma	- Vignette methodology - 1 ad hoc questionnaire investigating 4 items measuring victim blaming; 4 regarding criminality and impact of revenge pornography	Victimization was identified by participants as an offense that creates fear and pain.
Dardis, Davin, Lietzau, and Gidycz (2019) [39]	318 women	Public stigma	- Ad hoc questions asking who they disclosed their victimization experience to and how long after it happened; - Social Reactions Questionnaire [40]	- 92.6% of the participants who experienced Unwanted pursuit behaviors (UPBs) by an ex-partner disclosed the experience to one or more individuals; - 51.1% of the participants disclosed immediately after the victimization experience; - 11.3% of the participants disclosed 3 months or more after the victimization experience; - Reactions to the disclosure of women were generally more positive than negative; - The influence of UPBs on the development of PTSD was partially explained by increased negative social reactions.
DePrince, Welton-Mitchell, and Srinivas (2014) [41]	174 women	Public stigma	Short version of SRQ [40].	Symptoms of PTSD of a higher degree of severity (but not depression) after experiencing partner abuse contributed to negative social reactions 1 year later.
Doran and Hutchinson (2017) [42]	503 participants (86.7% were women) 13.1% were men	Public stigma	A questionnaire developed ad hoc with two sections: (1) Demographics; (2) 19 items exploring perceptions, knowledge, and attitudes of DV.	14% of the participants thought that it is justifiable to use physical force in intimate relationships.
Eigenberg and Policastro (2016) [27]	482 university students (43% were men and 57% were women)	Public stigma	- Awareness of IPV: Ad hoc questions to assess the effect of IPV education or training on respondents’ willingness to assume blame; - Experience with IPV: Ad hoc questions to investigate whether they, or a friend, or a family member had ever experienced IPV; - Justification: Ad hoc questions to investigate the justification of violence by participants in intimate relationships; - Attitudes towards women: Questions taken from the Ambivalent Sexism Inventory [43] regarding the equality of women within society; - General victim blaming scale: Ad hoc questions to investigate participants’ willingness to blame survivors in a broad sense; - Blaming the female survivor: Ad hoc questions to blame IPV survivors.	Participants were generally unwilling to blame female IPV survivors. However, higher levels of blame were present when survivors did not leave an abusive relationship.
Esposito, Di Napoli, Esposito, Carnevale, and Arcidiacono (2020) [44]	235 participants (57% were men and 43% were women)	Public stigma	A questionnaire was constructed ad hoc investigating the following aspects: Blame attribution; Strategies against violence; Demographic data.	The participants appeared to be able to propose an intervention to violence in relation to social factors.
Fleming and Franklin (2021) [45]	523 police personnel (89.1% were men and 10.9% women)	Public stigma	- Domestic Violence Myth Acceptance Scale [46]; - Family violence calls for service response in the previous 12 months measured with one ad hoc item; - Participant perceptions of preparedness to respond to family violence incidents were measured with a measured ad hoc item; - Prior specialized training was measured with three ad hoc items; - Attitudes Toward Crime Victims index [47]	- General low levels of IPV myth endorsement in the participants. - Prior general training was related to the feeling of preparedness to respond to family violence.
Flicker, Cerulli, Swogger, and Talbot (2012) [48]	131 women	Public and self-stigma	Women reported how frequently the person they talked about violence to (1) offered emotional support, (2) gave advice to break up with the batterer (3) or to stay with him, (4) blamed the survivor, (5) blamed the batterer, (6) offered help, or (7) continued to be friends with the batterer.	- Strategies of coping, such as disengagement, denial, and self-blame, were found to be associated with depression and post-traumatic symptoms. - Participants reported more supportive responses than non-supportive ones.
Gutowski and Goodman (2020) [49]	19 women	Public and self-stigma	Interview	- Women reported institutional betrayal and the experience of not being heard; - They also experienced economic, systemic, psychological, and relational barriers.
Halket, Gormley, Mello, Rosenthal, and Mirkin (2014) [50]	First study: 116 college students Second study: 183 college students	Public stigma	- Vignette methodology; - 14-question survey rating the survivor on her personality traits and the behavior of both the survivor and the batterer.	- The survivor was rated more positively when she left the abuser - Although education about the risks of violence is useful, it was not sufficient to change blame attitudes among young adults. It may be more effective for educators to challenge the attribution process instead.
Keller and Honea (2016) [51]	Interview: 13 women Focus Group: 22 individuals from the general population (11 were men and 11 were women)	Public and self-stigma	- In-depth interviews; - Four focus groups.	The themes emerged: Minimization of abuse; Victim blaming; Bystander helplessness; Abuse as a result of patriarchal relationships; Gender Backlash
Kim and Hogge (2015) [52]	152 women	Public stigma	- Vignette methodology - Ad hoc questions on participants’ acknowledgment of the abuse described in the vignettes and on help-seeking attitudes (i.e., how willing would they be to seek help from eight sources of support—e.g., informal support, counselling support, domestic violence and criminal justice services—if they were the survivor depicted in the vignettes).	- Participants were more likely to recognize physically violent acts as abuse. - Participants identified informal helpers as a preferred source of support compared to formal supporters
Meyer (2015) [53]	28 women	Public and self-stigma	Semi-structured interview	- 64.3% of the sample reported previous experiences with the victim-blaming attitude and the feeling of not being worth the help of supporters; - Survivors reported that they had to demonstrate that their suffering was not due to their own actions.
McKinley, Lilly, Knipp, and Liddell (2021) [54]	Qualitative data: 436 participants in total (254 individual interviews; 27 focus groups and 64 family interviews). Quantitative data: 127 participants	Public and self-stigma	Qualitative data: - The interviews used a life-history approach. Quantitative data: - Domestic Violence Blame Scale (DVBS) [55]	- Resilience was significantly negatively related to attitudes towards patriarchal gender roles and victim blame. - Participants with an experience of IPV victimization were more likely to endorse patriarchal gender role attitudes
Nikolova, Steiner, Postmus, Hetling, and Johnson (2021) [56]	237 women	Public stigma	New Jersey Assessment of Domestic Violence Risk and Impact (NJ-ADVRI) [57] which included questions on waiver recommendations	- Advocates recommended waivers more likely to survivors who experienced high levels of physical abuse and PTSD and left the abuser. - EA is recommended for women who have experienced emotional abuse or stalking to high or moderate degrees, and for women who have experienced moderate to low levels of stalking if they have children.
Overstreet, Willie, and Sullivan (2019) [14]	212 women	Public stigma	Social Reactions Questionnaire (SRQ; Ullman [40])	Stigmatizing public reactions to survivors’ disclosures of abuse experiences predicted depressive symptoms in IPV survivors, while general negative reactions (e.g., being angry with the perpetrator) did not.
Policastro and Payne (2013) [33]	370 students (65.4% of the sample were women)	Public stigma	An ad hoc questionnaire (10 items) investigating: - 5 items to measure participants’ level of myth acceptance and general misunderstandings about survivors; - 5 items to measure the acceptance of participants for penalties for survivors.	- 51% of the respondents believed that women do not leave abusive partners because they do not want to. - 42% of the participants agreed with the statement that battered women who remain with their abuser should have their children removed.
Riley and Yamawaki (2018) [58]	184 participants (108 were women and 76 were men)	Public Stigma	- Scenarios—A fictional scenario was used in which the survivor discloses her abuse to a friend; - Supportive Attitudes Toward Victim Scale (SAVS) (developed ad hoc); - Victim Blame Attribution (VBA) scale [59]	- Participants with higher values of benevolent sexism (BS) reported that the survivor should leave her abuser, or they would not provide any support. - Participants with higher values of BS and Right-Wing Authoritarianism (RWA) insisted that the wife should not anger the partner and that no one should interfere with the problems of a couple.
Rivera, Sullivan, and Zeoli (2012) [28]	19 women	Public and self-stigma	- Qualitative Interview (QI) - Ad hoc questionnaire: Court Mediator Experiences Survey (CMES) to evaluate mediators’ behaviors - Ad hoc questionnaire on secondary victimization	- 84% of the participants reported at least a negative experience in which they felt judged negatively by the mediator. - 63% of the participants reported previous experiences of secondary victimization characterized by feelings of blame, of being disbelieved, or dismissed. - 67% of the participants reported that they did not want to go back to court because they did not feel, for example, safe, believed, and respected.
Rollero and De Piccoli (2020) [26]	359 college students (76.5% were women)	Public stigma	Domestic Violence Myth Acceptance Scale [46]	- Acceptance of domestic violence myths was positively correlated with ambivalent sexism, the biological theory of gender and moral disengagement, and negatively with the social theory of gender. - The strongest predictors of adherence to domestic violence myths were Hostile Sexism (HS) and benevolence toward men (BM)
Sivagurunathan et al. (2019) [60]	189 hand therapists (HT) (89.2% were women and 10.8% were men)	Public stigma	- A 27-item questionnaire modified from 2 surveys previously published by Maiuro, et al. [61] and Connor, et al. [62]. - Participants’ experiences with IPV	- Low frequency of victim-blaming attitudes among HTs. - Participants agreed that dealing with IPV was consistent with their professional role. - Participants with previous experience with IPV were less likely to blame IPV survivors.
Srinivas and DePrince (2015) [63]	236 women	Self-stigma	Qualitative interview	- The negative response of the police was significantly related to PTSD; - Less perceived social support was associated with higher self-blame appraisals in relation to the IPV incident.
Woerner, Wyatt, and Sullivan (2019) [64]	173 Participants	Public stigma	- Semi-structured interview; - Social Reactions Questionnaire [40]	Three profiles emerged: One profile is characterized by negative reactions and few positive reactions to IPV disclosure. Participants had the highest levels of depression and PTSD. Another group had low levels of negative reactions and high levels of positive social reactions. The last group exhibited low levels of both negative and positive social reactions. No differences were found between groups 2 and 3 in the severity of mental health symptoms. Experience of negative reactions was related to more negative mental health.
Yamawaki, Ochoa-Shipp, Pulsipher, Harlos, and Swindler (2012) [65]	194 undergraduate students (77 were men and 117 were women)	Public stigma	- Vignette methodology - The Minimization Scale [59]; - Victim Blame Attribution Scale [59]; - Perpetrator Excuse Scale [59]; - Domestic Violence Myth Scale [65]	- The participants blamed the survivor in the vignette more when she returned to the batterer; - Negative attitudes toward IPV are influenced by myths about violence.

## Data Availability

Data sharing is not applicable to this article.

## Supplementary Materials

The following supporting information can be downloaded at: https://www.mdpi.com/article/10.3390/bs13030194/s1, PRISMA checklist; PRISMA_2020_abstract_checklist.
